# Degradation‐Resistant Hypoxia Inducible Factor‐2α in Murine Osteocytes Promotes a High Bone Mass Phenotype

**DOI:** 10.1002/jbm4.10724

**Published:** 2023-02-17

**Authors:** Sarah V. Mendoza, Deepa K. Murugesh, Blaine A. Christiansen, Zoe O. Genetos, Gabriela G. Loots, Damian C. Genetos, Clare E. Yellowley

**Affiliations:** ^1^ Department of Anatomy, Physiology, and Cell Biology, School of Veterinary Medicine University of California Davis Davis CA USA; ^2^ Lawrence Livermore National Laboratories Physical and Life Sciences Directorate Livermore CA USA; ^3^ Department of Orthopaedic Surgery University of California Davis Health Sacramento CA USA

**Keywords:** GENETIC ANIMAL MODELS, OSTEOCLAST, OSTEOCYTE

## Abstract

Molecular oxygen levels vary during development and disease. Adaptations to decreased oxygen bioavailability (hypoxia) are mediated by hypoxia‐inducible factor (HIF) transcription factors. HIFs are composed of an oxygen‐dependent α subunit (HIF‐α), of which there are two transcriptionally active isoforms (HIF‐1α and HIF‐2α), and a constitutively expressed β subunit (HIFβ). Under normoxic conditions, HIF‐α is hydroxylated via prolyl hydroxylase domain (PHD) proteins and targeted for degradation via Von Hippel‐Lindau (VHL). Under hypoxic conditions, hydroxylation via PHD is inhibited, allowing for HIF‐α stabilization and induction of target transcriptional changes. Our previous studies showed that *Vhl* deletion in osteocytes (*Dmp1‐cre; Vhl* ^*f/f*^) resulted in HIF‐α stabilization and generation of a high bone mass (HBM) phenotype. The skeletal impact of HIF‐1α accumulation has been well characterized; however, the unique skeletal impacts of HIF‐2α remain understudied. Because osteocytes orchestrate skeletal development and homeostasis, we investigated the role of osteocytic HIF‐α isoforms in driving HBM phenotypes via osteocyte‐specific loss‐of‐function and gain‐of‐function HIF‐1α and HIF‐2α mutations in C57BL/6 female mice. Deletion of *Hif1a* or *Hif2a* in osteocytes showed no effect on skeletal microarchitecture. Constitutively stable, degradation‐resistant HIF‐2α (HIF‐2α cDR), but not HIF‐1α cDR, generated dramatic increases in bone mass, enhanced osteoclast activity, and expansion of metaphyseal marrow stromal tissue at the expense of hematopoietic tissue. Our studies reveal a novel influence of osteocytic HIF‐2α in driving HBM phenotypes that can potentially be harnessed pharmacologically to improve bone mass and reduce fracture risk. © 2023 The Authors. *JBMR Plus* published by Wiley Periodicals LLC on behalf of American Society for Bone and Mineral Research.

## Graphical abstract

Osteocyte (OCY)‐specific degradation‐resistant HIF‐2α (HIF‐2α cDR) mice generated a high bone mass skeletal phenotype with increased trabecular bone and decreased cortical bone. Distal femoral metaphysis of HIF‐2α cDR mice showed evidence of bone marrow stromal tissue expansion, increased trabecular bone and new bone formation compared to *cre*‐negative control. Arrows indicate newly embedding osteocytes in newly forming bone. Created with BioRender.com.

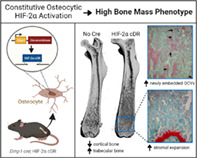

## Introduction

Molecular oxygen transforms energy into vital cellular processes that maintain cell function. It is an obligate component of mitochondrial respiration, amino acid catabolism, lipid metabolism, and other biochemical reactions essential for sustaining life.^(^ ^1^ ^)^ The bioavailability of molecular oxygen is dynamic, driven by blood flow and oxygen saturation.^(^ ^2^ ^)^ Pathophysiological insults such as vascular disease, heart failure, or fracture disrupt tissue perfusion, generating localized reductions in oxygen availability (hypoxia). Cells adapt to oxygen bioavailability via transcriptional changes that shift metabolism to anaerobic glycolysis, increase glucose uptake from the pericellular environment, and reestablish tissue vascularization to restore cellular and tissue function.^(^ ^3^ ^)^

Oxygen‐sensing transcription factors termed hypoxia‐inducible factors (HIFs) drive many, but not all, molecular responses to hypoxia. In the presence of sufficient molecular oxygen (normoxia), prolyl hydroxylase domain (PHD) proteins use oxygen as a cofactor to hydroxylate HIF‐α subunits at specific proline residues.^(^ ^4^ ^)^ Hydroxylated HIF‐α subunits are recognized by an E3 ligase complex Von Hippel‐Lindau (VHL) and are targeted for 26 S proteasomal degradation.^(^ ^5^ ^)^ Under hypoxic conditions, PHDs are unable to hydroxylate proline residues of HIF‐α subunits and HIF‐α escapes VHL recognition and subsequent degradation. HIF‐α binds to the stable β subunit (HIF‐β, or aryl hydrocarbon nuclear receptor [ARNT]) expressed in and confined to the nucleus.^(^ ^6^ ^)^ Three HIF‐α subunit isoforms exist: HIF‐1α, HIF‐2α, and HIF‐3α. HIF‐1α and HIF‐2α are transcriptional regulators, whereas HIF‐3α antagonizes HIF‐1α‐ and HIF‐2α‐mediated transcription.^(^ ^6^ ^)^ Thus, PHD isoforms and VHL mediate HIF‐α activity in response to changing oxygen levels.

HIF‐1α and HIF‐2α are highly homologous, both in structure and repertoire of transcriptional outcomes, yet their functions only partially overlap.^(^ ^7^ ^)^ Both HIF‐1α and HIF‐2α induce *Vegf* (angiogenesis) and *Glut1* (increase pericellular glucose uptake).^(^ ^3^ ^)^ HIF‐1α induces glycolytic enzymes and pH regulatory protein expression whereas HIF‐2α promotes genes associated with vascular invasion.^(^ ^3^ ^)^ Further, HIF‐1α and HIF‐2α expression and function vary in response to hypoxia. HIF‐2α is hydroxylated less efficiently by PHD proteins, resulting in stabilization at higher oxygen tensions compared to HIF‐1α.^(^ ^8^ ^)^ Therefore, HIF‐1α mediates acute, and HIF‐2α mediates chronic, response to hypoxia. HIF‐1α and HIF‐2α are composed of structurally and functionally similar transactivation domains at their C termini, DNA binding and dimerization domains at their N termini, and isoform specific N‐terminal transactivation domains (N‐TAD).^(^ ^7^ ^)^ Structural differences in the N‐TAD of HIF‐1α or HIF‐2α impart target gene specificity, because replacement of HIF‐2α N‐TAD with HIF‐1α N‐TAD is sufficient to confer HIF‐1α‐specific functionality to HIF‐2α.^(^ ^7^ ^)^ It is currently unknown which HIF‐α that isoforms osteocytes, the master orchestrators of skeletal activity,^(^ ^9^ ^)^ utilize to influence skeletal remodeling.

Osteogenesis and angiogenesis are intimately linked. Embryogenesis occurs in a hypoxic environment, driven by transcriptional changes induced by HIF‐α signaling that persist after cardiovascular development.^(^ ^10^ ^)^ During skeletal morphogenesis, vascular endothelial growth factor (VEGF) serves as a key facilitator of endochondral ossification in long bones via VEGF‐dependent vascularization.^(^ ^11^ ^)^ In this process, mesenchymal stem cells condense and differentiate into chondrocytes, proliferate, then form the cartilaginous blueprint for future bone replacement. Proliferating chondrocytes undergo hypertrophy and calcify surrounding avascular cartilage, creating a hypoxic environment. Hypertrophic chondrocytes express high levels of VEGF that induce vascular invasion which supplies nutrients and osteoprogenitors to support avascular cartilage replacement with vascularized bone wherein osteoclasts resorb calcified cartilage and osteoblasts form new bone. Thus, HIF‐α signaling is essential in the formation and maintenance of bone.

HIF‐α isoforms are differentially required in osteogenic cells. Single deletions of *Hif1a* or *Hif2a* in osteoprogenitors (*Osx‐cre*) had no effect on trabecular microarchitecture; however, dual deletion of HIF‐α isoforms (*Osx‐cre; Hif1a* ^*f/f*^ *; Hif2a* ^*f/f*^) reduced trabecular bone volume fraction.^(^ ^12^, ^13^ ^)^ *Hif1a* deletion in mature osteoblasts (*Bglap‐cre; Hif1a* ^*f/f*^) resulted in a moderate trabecular and cortical bone phenotype.^(^ ^14^, ^15^ ^)^ Our recent studies have shown that *Hif1a* deletion in osteocytes does not elicit a bone phenotype, shifting focus onto HIF‐2α as the possible dominant isoform responsible for driving osteocyte‐mediated changes in the skeleton.

The VHL/HIF system is highly active in osteocytes, which are the most abundant cell type in bone, comprising nearly 90% to 95% of adult bone cells.^(^ ^9^ ^)^ Osteocytes are embedded within mineralized matrix of both cortical and trabecular bone and can persist for decades.^(^ ^16^ ^)^ These long‐lived cells are hailed as the master regulator of bone remodeling due to their orchestration of osteoblast and osteoclast activity via paracrine secretion of growth factors and cytokines,^(^ ^17^ ^)^ and direct cell‐cell communication via gap junctions.^(^ ^18^ ^)^ In addition, osteocytes serve essential roles in mechanosensing, mineral homeostasis, and hematopoiesis.^(^ ^19^ ^)^ Due to the osteocyte's fundamental role in maintaining skeletal homeostasis, we sought to elucidate osteoanabolic potential of VHL/HIF signaling mechanism in these cells. Because they share genetic and pathophysiological similarities to humans, our studies utilize transgenic mouse models to discover skeletal effects of HIF‐α isoform manipulation. We previously showed that *Vhl* deletion in osteocytes increases HIF‐1α and HIF‐2α activity, which in turn generates dramatic increases in both cortical and trabecular microarchitecture compared to age‐matched wild‐type mice.^(^ ^20^ ^)^ To further investigate the role of osteocytic VHL/HIF signaling in driving high bone mass (HBM) phenotypes, we used osteocyte‐specific loss‐of‐function and gain‐of‐function HIF‐1α and HIF‐2α mutations to determine which isoform predominantly drives this phenotype.

## Materials and Methods

### Mice

*Vhl* ^*f/f*^ (#004081), *Hif1a* ^*f/f*^ (#007561), *Hif2a* ^f/f^ (#008407), LSL‐HIF1α^DPA^ (#009673), and LSL‐HIF2α^DPA^ (#009674) C57BL/6 mice were purchased from The Jackson Laboratory (Bar Harbor, ME, USA) and bred with mice expressing *cre* recombinase from the 10‐kilobase (kb)‐Dmp1 promoter (*Dmp1‐cre*) [B6N.FVB‐Tg (*Dmp1‐cre*)1Jqfe/BwdJ] (kindly provided by Dr. Jian Q. Feng, Department of Biomedical Sciences, Texas A&M University Baylor College of Dentistry). These matings produced progeny with osteocyte‐enriched deletion of *Vhl* (*Vhl* conditional knockout [cKO]), *Hif1a* (*Hif1a* cKO), and *Hif2a* (*Hif2a* cKO) or mice with osteocyte‐enriched degradation resistant HIF‐1α (HIF‐1α cDR) and HIF‐2α (HIF‐2α cDR).^(^ ^21^ ^)^ HIF‐α cDR mutant protein escapes hydroxylation by PHD proteins via mutation of the two target hydroxylation residues from proline to alanine. HIF‐α cDR mutant protein cannot be recognized and polyubiquitinated by VHL protein, evading proteasomal degradation, resulting in constitutive activation of HIF‐α signaling regardless of oxygen tension. Mice were group‐housed, provided water and standard chow *ad libitum*, then euthanized at 16 weeks. All animal experiments were approved by the Lawrence Livermore National Laboratory and University of California, Davis Institutional Animal Care and Use Committee and conformed to the Guide for the care and use of laboratory animals.

### Histology and histomorphometry

Right femurs from female mice were dissected at euthanasia, fixed, decalcified, processed, and embedded as described.^(^ ^22^ ^)^ Using a steel blade mounted on a rotary microtome (Microm HM355S, Thermo Fisher Scientific, Kalamazoo, MI, USA), longitudinal sections were cut into 5‐μm slices for all histological staining. Hematoxylin/eosin and Alcian blue staining were performed as in Toupadakis and colleagues.^(^ ^23^ ^)^ Safranin O and Fast green staining were performed as in Tran and colleagues.^(^ ^24^ ^)^ Tartrate‐resistant acid phosphatase (TRAP) staining was performed as per the University of Rochester Medical Center: Center for Musculoskeletal Research's “TRAP Stain for Paraffin Sections” protocol.^(^ ^25^ ^)^ All bright‐field images were taken with a Retiga 1300 camera (QImaging, Surrey, BC, Canada) via NIS Elements v2013 acquisition software (Nikon, Melville, NY, USA) on a Nikon Eclipse E600 (Nikon, Melville, NY, USA) microscope. Quantitative histomorphometry was performed using the Bioquant Osteo 2014 (BioQuant, Nashville, TN, USA) histomorphometry system. Osteoclast number over bone surface was measured from a 2‐mm^2^ region of interest (ROI), 25–30 μm from the most proximal point of the growth plate for each animal. Fiji ImageJ was used to measure bone marrow adiposity.^(^ ^26^ ^)^ A 10‐mm^2^ ROI extending from the proximal boundary of the growth plate was used to measure adipocyte surface area and total surface area, calculating bone marrow adiposity as a percentage for each animal.

### Micro‐computed tomography

At euthanasia, the left femur was extracted, placed in 10% neutral‐buffered formalin for 48 hours, then stored in 70% ethanol at 4°C. A 1.5‐mm span of the distal femoral metaphysis was scanned with micro‐computed tomography (μCT) (μCT 35; Scanco Medical AG, Brüttisellen, Switzerland) at 6‐μm resolution using 55‐kV peak tube potential and 300‐ms integration time to measure trabecular three‐dimensional morphometric properties of metaphyseal trabecular bone as described.^(^ ^27^, ^28^ ^)^ Extensive metaphyseal trabecularization made it difficult to determine the endocortical shell boundary from trabecular bone of HBM femurs. To address this, all bone within the metaphyseal region—including the cortical shell—was measured to determine total bone volume fraction within the trabecular compartment of HIF2α cDR and *Vhl* cKO mice (Fig. S1). A 0.6‐mm span of the middle femoral diaphysis was scanned with parameters previously described to measure cortical three‐dimensional morphometric properties. Extensive cortical porosity made it difficult to determine cortical thickness and medullary area of HBM femurs. To address this, ROIs including dense and porous cortical bone were used to determine cortical thickness (Fig. S2A), and a single endocortical boundary was used to determine medullary area (Fig. S2B). To address the quality/continuity of the cortical bone on the interior of or cortical measurements, we also measured cortical porosity.

### Statistical analysis

All data are expressed as the mean ± standard deviation (SD). Unpaired Student's *t* test, or multiple comparison one‐way analysis of variance (ANOVA) followed by Fisher's least significant difference (LSD) test were used where appropriate, using GraphPad Prism version 9.4.1 (681) for Windows (GraphPad Software, San Diego, CA, USA). For all tests, *p* value <0.05 was considered statistically significant. Groups with different letters are statistically different from each other.

## Results

### Deletion of neither osteocytic HIF‐1α nor HIF‐2α elicits a skeletal phenotype

We previously demonstrated that osteocyte‐specific deletion of *Vhl* produced a high‐bone mass phenotype, whereas an inverse phenotype was not observed in mice lacking osteocytic *Hif1a*.^(^ ^20^ ^)^ This outcome suggested that HIF‐1α is dispensable for osteocyte regulated skeletal phenotype and that osteocytes preferentially utilize HIF‐2α instead. Previous studies deleting *Hif2a* in mature osteoblasts reported impairment of osteoblast differentiation resulting in a low bone mass phenotype.^(^ ^29^ ^)^ To examine the requirement of HIF‐2α in osteocytes, we used a cre‐loxP system where *cre* recombinase was expressed under the control of the 10‐kb‐Dmp1 promoter.^(^ ^30^ ^)^ Mice were born at expected Mendelian frequency and showed no overt phenotype at 16 weeks of age.

**Fig. 1:**
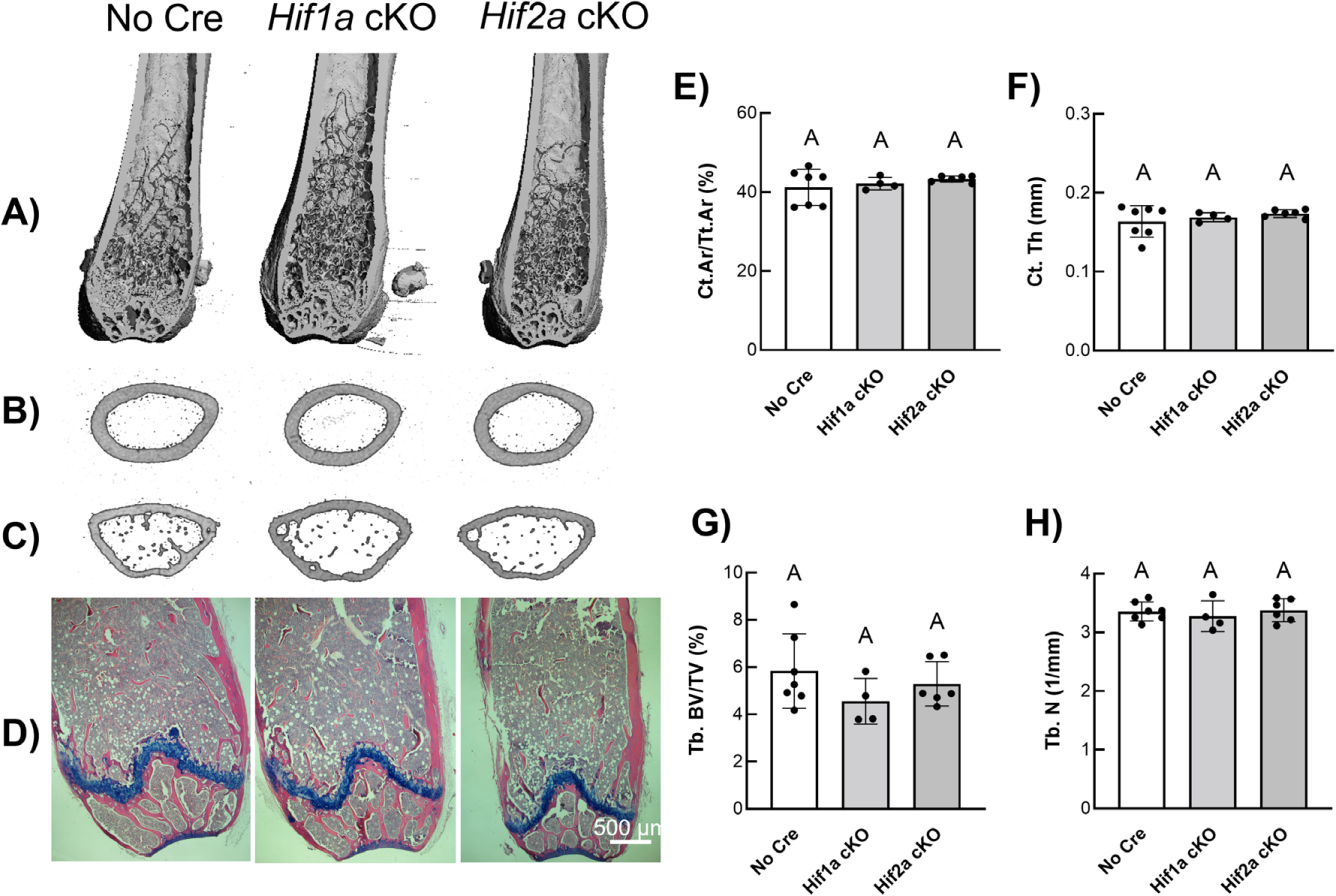
Osteocyte specific *Hifa* isoform deletion does not elicit a skeletal phenotype. Representative longitudinal μCT images of distal femur (*A*) and representative transverse images from the femoral midshaft (*B*) and femoral metaphysis (*C*). (*D*) Representative hematoxylin/eosin and Alcian blue staining of distal femurs. Quantitative cortical microarchitecture measurements of Ct.Ar/Tt.Ar (*E*) and Ct.Th (*F*) from the femoral mid‐diaphysis. Quantitative trabecular microarchitecture measurements of Tb.BV/TV (*G*) and Tb.N (*H*) from the distal femoral metaphysis from *cre*‐negative, *Hif1a* cKO, and *Hif2a* cKO mice. Bars represent mean ± SD; *n* = 4–7 female mice per genotype; groups with different letters are statistically different from each other. Ct.Ar/Tt.Ar = cortical bone area fraction; Ct.Th = cortical thickness; Tb.BV/TV = trabecular bone area fraction; Tb.N = trabecular number.

Representative longitudinal μCT images of the distal femur (Fig. 1*A*) and representative transverse images of the femoral midshaft (Fig. 1*B*) and femoral metaphysis (Fig. 1*C*) revealed no gross changes in skeletal microarchitecture in *Hif1a* cKO and *Hif2a* cKO mice compared to *cre*‐negative control mice (Fig. 1*A*). Hematoxylin/eosin and Alcian blue staining of distal femoral epiphysis (Fig. 1*D*) showed no significant gross changes in bone morphology in *Hif1a* cKO and *Hif2a* cKO mice compared to *cre*‐negative control mice (Fig. 1*D*). Quantitative microarchitecture measurements of cortical bone area fraction (Ct.Ar/Tt.Ar) (Fig. 1*E*) and cortical thickness (Ct.Th) (Fig. 1*F*) from the femoral mid‐diaphysis of *Hif1a* cKO and *Hif2a* cKO mice were indistinguishable from *cre‐*negative control femurs. Quantitative microarchitecture measurements of trabecular bone area fraction (Tb.BV/TV) (Fig. 1*G*) and trabecular number (Tb.N) (Fig. 1*H*) from the distal femoral metaphysis of *Hif1a* cKO, and *Hif2a* cKO mice were indistinguishable from *cre*‐negative control femurs. Additional quantitative cortical and trabecular microarchitecture parameters listed in Table 1 further demonstrated that single *Hif1a* or *Hif2a* deletion had no significant effect on skeletal phenotype when compared to *cre*‐negative control mice.

**Table 1:** Trabecular and Cortical Microarchitecture Are Unaffected by Osteocytic *Hifa D*eletion

Parameter	No Cre (*n* = 7)	*Hif1a* cKO (*n* = 4)	*Hif2a* cKO (*n* = 6)
Cortical microarchitecture			
Me.Ar (cm^2^)	1.035 ± 0.130	1.036 ± 0.048	1.026 ± 0.035
Ct.Po (%)	7.979 ± 0.0684	7.995 ± 0.239	7.638 ± 0.213
Ct.TMD (mg HA/cm^3^)	1193.006 ± 25.541	1134.392 ± 1.104	1181.085 ± 55.401
Trabecular microarchitecture			
Tb.Th (mm)	0.038 ± 0.004	0.036 ± 0.002	0.038 ± 0.001
Tb.Sp (mm)	0.297 ± 0.014	0.305 ± 0.024	0.296 ± 0.019
Tb.BMD (mg HA/cm^3^)	1046.735 ± 115.706	932.827 ± 8.031	961.653 ± 27.309

*Note*: Values are shown as mean ± SD; *n* = 4–7 mice per genotype. Quantitative cortical microarchitecture measurements of Me.Ar, Ct.Po, and Ct.TMD from the femoral mid‐diaphysis of *cre*‐negative, *Hif1a* cKO, and *Hif2a* cKO mice. Quantitative trabecular microarchitecture measurements of Tb.Th, Tb.Sp, and Tb.BMD from the distal femoral metaphysis of *cre*‐negative, *Hif1a* cKO, and *Hif2a* cKO mice.

Abbreviations: Ct.Po, cortical porosity; Ct.TMD, cortical mineralized density; HA, hydroxyapatite; Me.Ar, medullary area; Tb.BMD, trabecular mineralized density; Tb.Sp, trabecular separation; Tb.Th, trabecular thickness.

### Osteocyte‐specific degradation‐resistant HIF‐2α accumulation generates a HBM phenotype

To determine which HIF‐α isoform mediates the HBM phenotype observed in *Vhl* knockout mice, we used transgenic mice with osteocyte specific gain‐of‐function HIF‐1α or HIF‐2α mutations. These mice possess nucleotide substitutions at two proline residues (HIF‐1α: P402A, P564A; HIF‐2α: Pro‐405A, Pro‐531A) resulting in the replacement of proline by alanine residues (termed dPA; delta Proline to Alanine substitution) at the HIF‐α hydroxylation site, preventing oxygen‐dependent prolyl hydroxylation via PHDs and subsequent VHL recognition and degradation. HIF‐1α dPA^f/f^ and HIF‐2α dPA^f/f^ mice containing mutant human complementary DNA (cDNA) encoding HIF‐1α dPA and HIF‐2α dPA protein were knocked into the ROSA26 locus preceded by a lox‐stop‐lox (LSL) cassette.^(^ ^30^ ^)^ Upon *cre*‐mediated recombination, the STOP sequence is removed, allowing for expression of the mutated coding sequence.^(^ ^30^ ^)^ LSL‐HIF‐1α dPA mice will hereafter be called HIF‐1α conditionally‐degradation resistant (HIF‐1α cDR), and LSL‐HIF‐2α dPA mice will be referred to as HIF‐2α conditionally‐degradation resistant (HIF‐2α cDR). Immunohistochemical (IHC) analysis of femoral cortical osteocytes confirmed increased HIF‐1α expression in HIF‐1α cDR mice and reduced expression in *Hif1a* cKO mice (Fig. S3). We also demonstrate increased HIF‐2α expression in HIF‐2α cDR mice; expression of HIF‐2α was not detected in *cre*‐negative and *Hif2a* cKO osteocytes by IHC (Fig. S4).

**Fig. 2:**
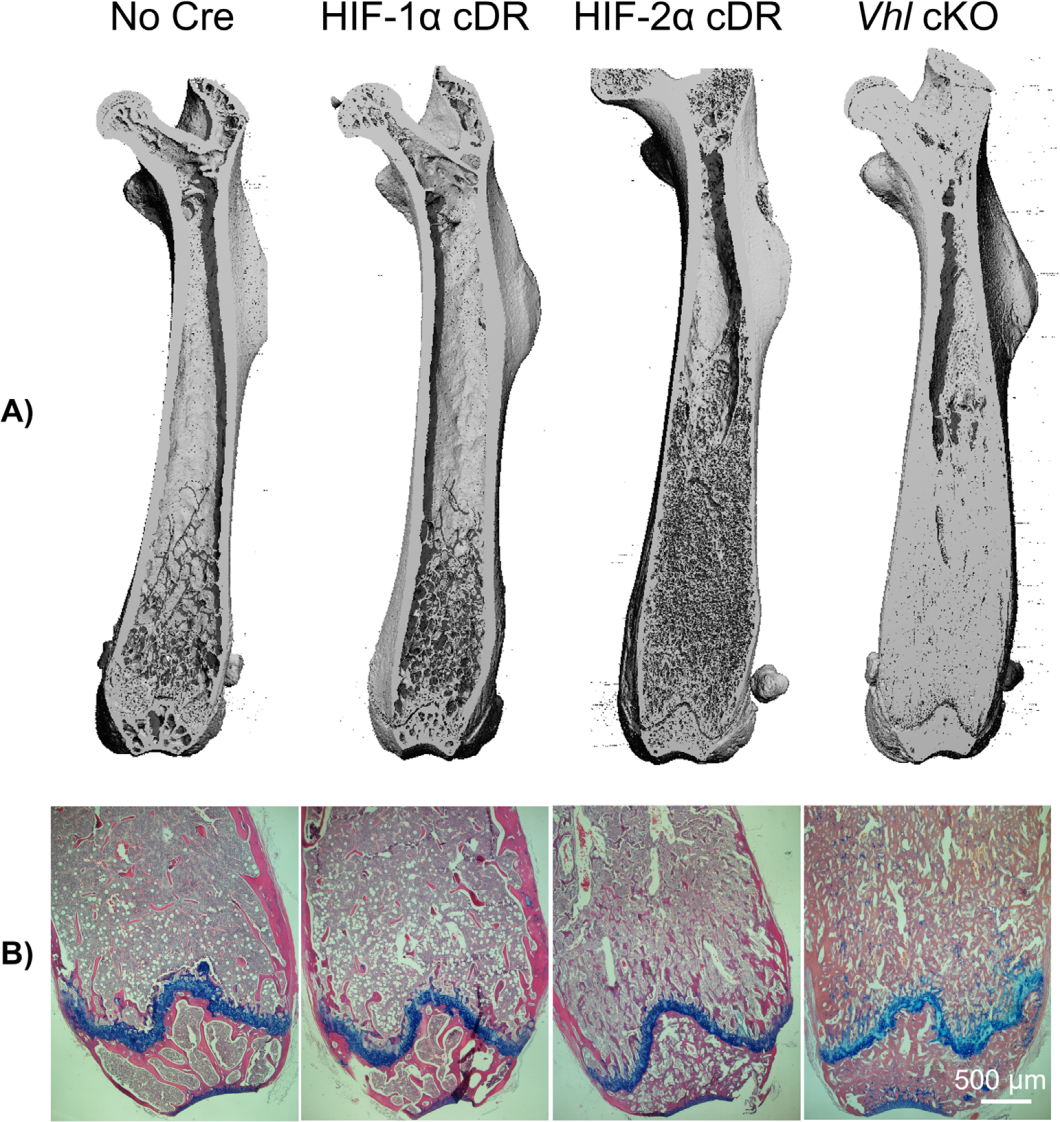
Osteocyte‐specific degradation‐resistant HIF‐2α accumulation generates an HBM phenotype. Representative 3D reconstruction images of distal femurs (*A*) and hematoxylin/eosin and Alcian blue staining of distal femoral epiphysis (*B*) of *cre*‐negative, HIF‐1α cDR, HIF‐2α cDR, and *Vhl* cKO mice.

μCT analysis was performed to evaluate skeletal phenotype. Longitudinal femoral reconstruction images showed no gross changes in skeletal development of HIF‐1α cDR mice compared to *cre*‐negative control mice (Fig. 2*A*). HIF‐2α cDR femurs showed an increase in trabecular bone compared to HIF‐1α cDR and *cre‐*negative control mice (Fig. 2*A*). However, increases in trabecular bone of HIF‐2α cDR femurs were less robust compared to *Vhl* cKO femurs (Fig. 2*A*). Hematoxylin/eosin and Alcian blue staining demonstrated a reduction in hematopoietic tissue and an increase in both trabecular bone and stromal tissue in the bone marrow space of HIF‐2α cDR mice compared to HIF‐1α cDR and *cre‐*negative control mice (Fig. 2*B*). In contrast, the bone marrow cavity of *Vhl* cKO femurs demonstrated dense trabeculated bone with small spaces filled with vasculature and hematopoietic tissue. (Fig. 2*B*).

**Fig. 3:**
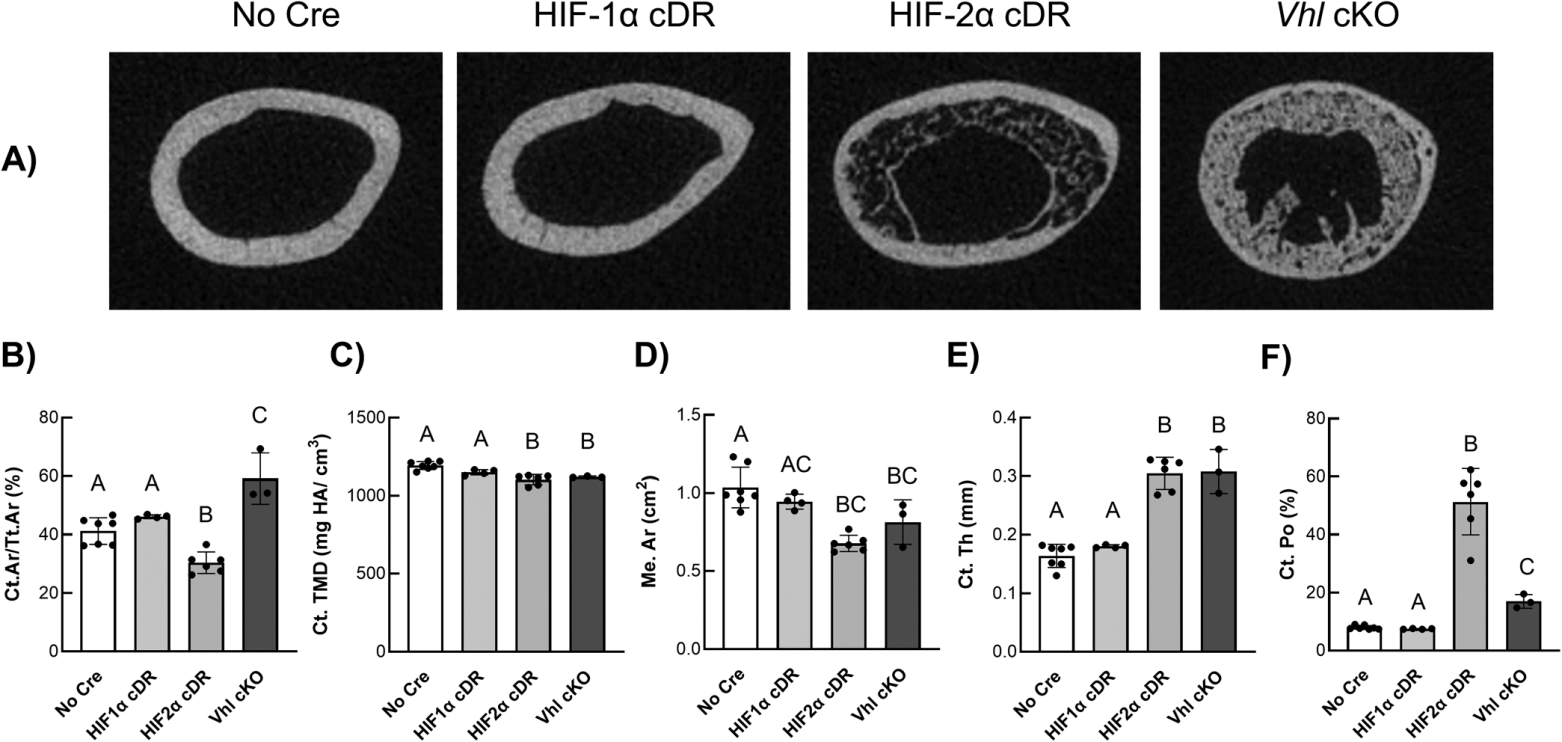
Osteocyte‐specific HIF‐2α accumulation influences cortical microarchitecture. (*A*) Representative μCT transverse images of mid‐diaphyseal cortical microarchitecture from *cre*‐negative, HIF‐1α cDR, HIF‐2α cDR, and *Vhl* cKO femurs. Quantitative cortical microarchitecture measurements of Ct.Ar/Tt.Ar (*B*), Ct.TMD (*C*), Me.Ar (*D*), Ct.Th (*E*), and Ct.Po (*F*) of femoral mid‐diaphysis from *cre*‐negative (*n* = 7), HIF‐1α cDR (*n* = 4), HIF‐2α cDR (*n* = 6), and *Vhl* cKO (*n* = 3) femurs. Bars represent mean ± SD; groups with different letters are statistically different from each other. Ct.Ar/Tt.Ar = cortical bone area fraction; Ct.Po = cortical porosity; Ct.Th = cortical thickness; Ct.TMD = cortical tissue mineral density; Me.Ar = medullary area.

Representative transverse μCT images from the mid‐femoral diaphysis of HIF‐2α cDR mice showed significant cortical thickening of the femoral midshaft (Fig. 3*A*). Quantitative cortical microarchitecture measurements of HIF‐2α cDR femurs showed significantly decreased cortical bone area fraction (Fig. 3*B*), cortical tissue mineral density (Ct.TMD) (Fig. 3*C*), and medullary area (Me.Ar) (Fig. 3*D*) yet increased cortical thickness (Fig. 3*E*) compared to *cre*‐negative control mice. HIF‐2α cDR mice also revealed robust increases in cortical porosity (Ct.Po) (Fig. 3*F*). In contrast, *Vhl* cKO mice exhibited a significant increase in cortical bone area fraction (Fig. 3*B*) when compared to *cre*‐negative control mice. Cortical tissue mineral density in *Vhl* cKO mice (Fig. 3*C*) was significantly decreased compared to *cre*‐negative mice but was similar to HIF‐2α cDR mice. *Vhl* cKO medullary area (Fig. 3*D*) was significantly reduced compared to *cre*‐negative mice, whereas cortical thickness (Fig. 3*E*) was significantly increased. However, medullary area and cortical thickness were similar to that of HIF‐2α cDR mice. Cortical porosity (Fig. 3*F*) of *Vhl* cKO femurs were significantly increased compared to *cre*‐negative control but significantly lower than HIF‐2α cDR mice. Cortical microarchitecture of HIF‐1α cDR femurs was statistically indistinguishable from that of *cre*‐negative controls.

**Fig. 4:**
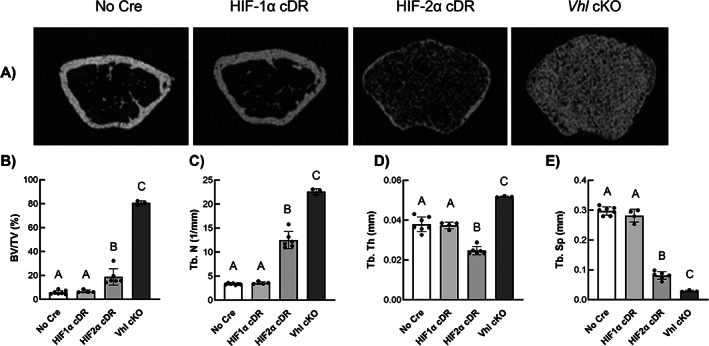
Osteocyte‐specific HIF‐2α accumulation augments trabecular microarchitecture. (*A*) Representative μCT transverse images of metaphyseal trabecular microarchitecture from *cre*‐negative, HIF‐1α cDR, HIF‐2α cDR, and *Vhl* cKO femurs. Quantitative trabecular microarchitecture measurements of the trabecular compartment BV/TV (*B*), Tb.N (*C*), Tb.Th (*D*) and Tb.Sp (*E*) of the distal femoral metaphysis from *cre*‐negative (*n* = 7), HIF‐1α cDR (*n* = 4), HIF‐2α cDR (*n* = 6), and *Vhl* cKO (*n* = 3) femurs. Bars represent mean ± SD; groups with different letters are statistically different from each other. BV/TV = bone volume fraction; Tb.N = trabecular number; Tb.Sp = trabecular separation; Tb.Th = trabecular thickness.

In the distal femoral metaphysis, HIF‐2α cDR mice revealed significant increases in bone volume fraction of the trabecular compartment (Fig. 4*A*, 4*B*) and trabecular number (Fig. 4*C*) with concomitant decreases in trabecular thickness (Fig. 4*D*) and separation (Fig. 4*E*) when compared to *cre*‐negative controls. These outcomes were phenocopied in *Vhl* cKO animals except for trabecular thickness (Fig. 4*D*), which was significantly increased compared to all other genotypes. HIF‐1α cDR mice showed no differences in trabecular microarchitecture compared to *cre*‐negative control mice.

### Osteocyte specific HIF‐2α accumulation drives distal trabecularization of the bone marrow compartment at the expense of hematopoietic tissue

**Fig. 5:**
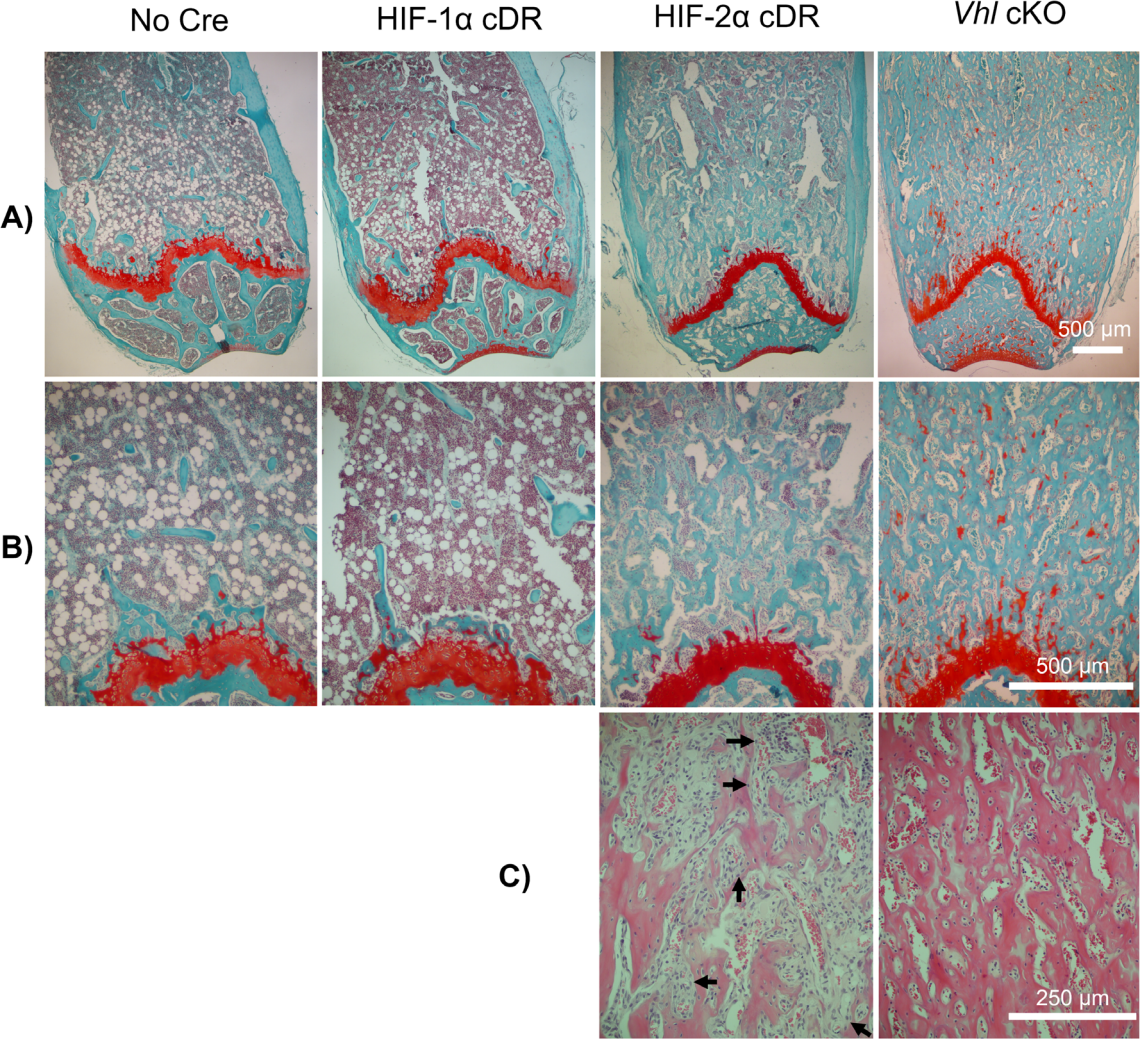
Osteocyte‐specific HIF‐2α accumulation drives distal trabecularization of the bone marrow compartment at the expense of hematopoietic cell lineages. Representative Safranin‐O/Fast green staining of the distal femoral epiphysis at 4× (*A*) and 10× (*B*) of *cre*‐negative, HIF‐1α cDR, HIF‐2α cDR, and *Vhl* cKO. (*C*) Representative hematoxylin/eosin and Alcian blue staining of bone marrow compartment of HIF‐2α cDR and *Vhl* cKO femurs. Arrows indicate newly embedding osteocytes in newly forming bone.

We considered that the observed increase in bone mass of HIF‐2α cDR mice may result from calcified cartilage that was retained during development, which can be visualized with Safranin O. No staining of retained cartilage was observed in the distal femoral metaphysis of HIF‐1α cDR, HIF‐2α cDR, and *cre*‐negative control mice, but was present, to a small extent, in the distal femoral metaphysis of *Vhl* cKO mice (Fig. 5*A*,*B*).

Histology of the distal metaphysis of the HIF‐2α cDR femur revealed significant expansion of bone marrow stroma and a clear reduction in hematopoietic tissue when compared to *cre*‐negative controls (Fig. 5*B*,*C*). Numerous bony spicules developing in the expansive stroma of HIF‐2α cDR mice and the appearance of embedding osteocytes was suggestive of intramembranous bone formation (Fig. 5*C*). In contrast, and as described,^(^ ^20^ ^)^ the *Vhl* cKO distal femoral metaphysis demonstrated thick abundant trabecularized bone with numerous spaces filled with vasculature and few bone marrow hematopoietic cells (Fig. 5*C*). No increase in bone marrow stroma was evident in *Vhl* cKO mice or HIF‐1α cDR femurs. This suggests that the expansion of bone marrow stroma is unique to mice with osteocytic HIF‐2α accumulation. No significant changes in bone marrow adiposity were observed between HIF‐1α cDR and *cre*‐negative controls; however, reductions were observed in the HIF‐2α cDR and *Vhl* cKO mice (Fig. S5), most likely the result of bone marrow displacement by marked increases in bone mass.

**Fig. 6:**
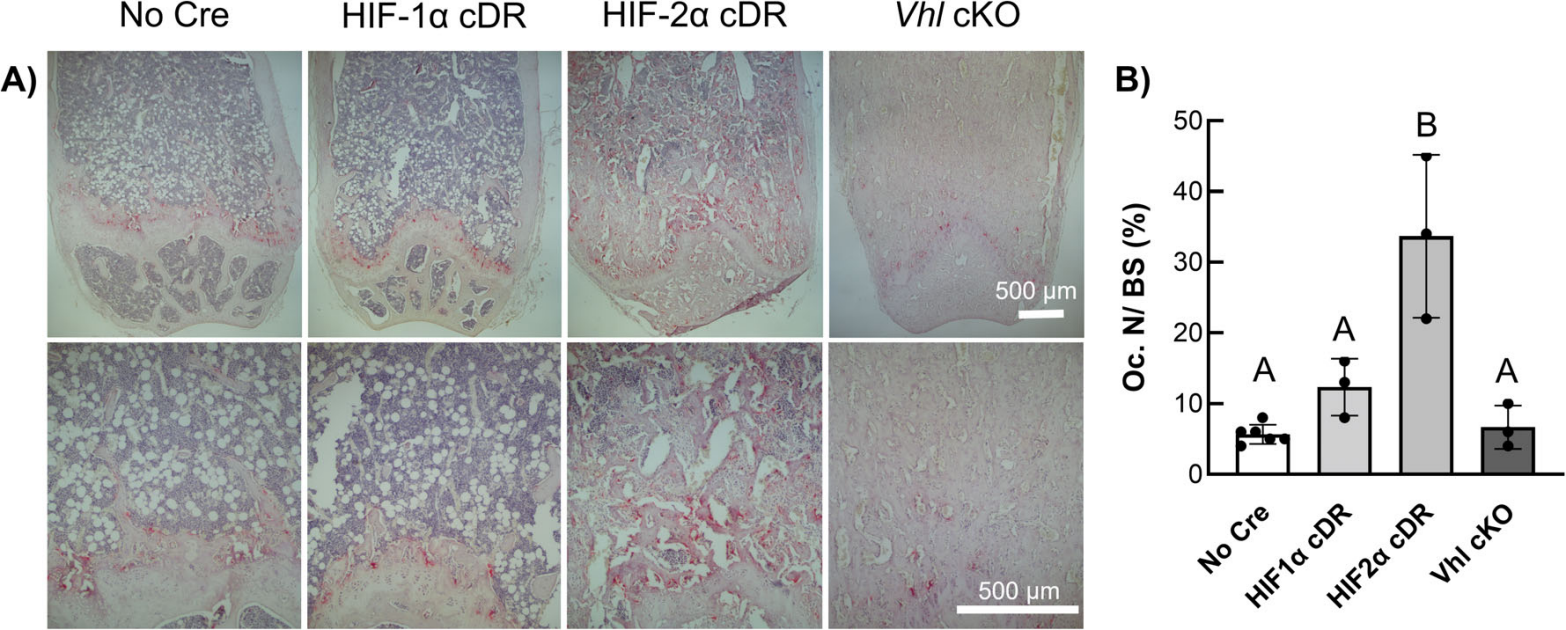
Osteocyte‐specific HIF‐2α accumulation increases osteoclast cell number. Representative TRAP‐staining of osteoclasts at 4× (top row) and 10× (bottom row) (*A*) and quantification of Oc.N/BS (*B*) in *cre*‐negative (*n* = 5), HIF‐1α cDR (*n* = 3), HIF‐2α cDR (*n* = 3), and *Vhl* cKO (*n* = 3) distal femoral epiphysis. Bars represent mean ± SD; groups with different letters are statistically different from each other. Oc.N/BS = osteoclast number over bone surface.

TRAP staining of longitudinal distal femoral sections revealed increased osteoclast staining in HIF‐2α cDR mice compared to *cre*‐negative, HIF‐1α cDR, and *Vhl* cKO mice (Fig. 6*A*). Histomorphometric measurements revealed a significant increase in osteoclast number over bone surface (Oc.N/BS) in HIF‐2α cDR mice compared to *cre*‐negative control, HIF‐1α cDR, and *Vhl* cKO mice (Fig. 6*B*).

## Discussion

HIF‐α isoforms are differentially required among osteoprogenitors, pre‐osteoblasts, and mature osteoblasts; however, HIF‐α isoform requirement in osteocytes remains understudied. Herein, we demonstrated that deletion of either *Hif1a* or *Hif2a* in osteocytes showed no significant effect on skeletal microarchitecture, resulting in a skeletal phenotype indistinguishable from the *cre*‐negative control (Fig. 1), suggesting compensatory mechanisms involving functional redundancy driven by either HIF‐α isoform, or interference from parallel *Vhl*‐driven yet HIF‐independent pathways. The lack of phenotype we observed with *Hif1a* or *Hif2a* deletion in osteocytes contrasts what has been previously shown when deletions are mediated earlier in the lineage. Deletion of *Hif1a* in limb bud mesenchymal progenitors (*Prx1‐cre*) resulted in massive chondrocyte death and severe limb abnormalities.^(^ ^12^ ^)^ Conversely, *Hif2a* deletion via *Prx1‐cre* generated a HBM phenotype characterized by increases in trabecular and cortical bone without changes in osteoclast number^(^ ^12^ ^)^; suggesting that *Hif2a* deletion negatively affects bone formation, not bone resorption in pre‐osteoblasts and their descendants. In committed osteoprogenitors, single deletion of *Hif1a* or *Hif2a* (*Osx‐cre*) had no effect on trabecular microarchitecture, whereas dual deletion of *Hif1a* and *Hif2a* (*Osx‐cre; Hif1a* ^*f/f*^ *; Hif2a* ^*f/f*^) decreased trabecular bone volume fraction.^(^ ^13^, ^31^ ^)^ Therefore, both HIF‐α isoforms are required for determining skeletal phenotype in mesenchymal osteoprogenitors and pre‐osteoblasts. Taken together, *Hif2a* deletion leaves open the opportunity for compensatory signaling by HIF‐1α. In contrast, HIF‐2α cDR generates a state of robust and persistent HIF‐2α expression and thus, HIF‐2α‐driven transcription.

*Hif1a* deletion in mature osteoblasts (*Bglap‐cre; Hif1a* ^*f/f*^) decreased trabecular bone volume fraction, cortical cross‐sectional area, and vascularity.^(^ ^14^, ^15^ ^)^ Similar changes in cortical and trabecular phenotype, although more moderate, were observed in mice lacking osteoblastic *Hif2a* (*Bglap‐cre; Hif2a* ^*f/f*^) whereas they maintained reduced vascular density.^(^ ^14^, ^15^ ^)^ Thus, although osteogenesis and angiogenesis are often intimately linked, there are exceptions, suggesting *Hif2a*‐driven uncoupling of angiogenesis from osteogenesis in osteoblasts. Dual deletion of osteoblastic *Hif1a* and *Vhl* (*Bglap‐cre; Hif1a* ^*f/f*^ *; Vhl* ^*f/f*^) generated a HBM phenotype less severe than *Vhl* deletion alone (*Bglap‐cre; Vhl* ^*f/f*^),^(^ ^14^ ^)^ suggesting that HIF‐1α and HIF‐2α influence skeletal phenotype in osteoblasts.

*Hif2a* transcript levels increase during osteoblastic differentiation, wherein HIF‐2α downregulates osteocalcin (OCN) and RUNX2 (known osteoblast differentiation markers^(^ ^32^ ^)^) via *Twist2*.^(^ ^29^ ^)^ Osteoblastic receptor activator of nuclear factor kappa‐β ligand (RANKL), known to drive osteoclastogenesis, increases concomitantly with HIF‐2α during osteoblast differentiation, resulting in increased osteoblast‐mediated osteoclastogenesis and subsequent bone resorption.^(^ ^29^ ^)^ Mice with heterozygous *Hif2a* deletion in osteoblasts (*Col1a1‐cre; Hif2a* ^*+/f*^) showed significant increases in trabecular bone due to enhanced osteoblast differentiation and reduced osteoclastogenesis; no cortical phenotype was observed.^(^ ^29^ ^)^ Like mesenchymal progenitor HIF‐2α, osteoblastic HIF‐2α negatively regulates bone formation while promoting bone resorption. Our results differentiate state‐dependent contributions of HIF‐2α toward skeletal phenotype, wherein HIF‐2α stabilization in osteocytes promotes osteoanabolism.

Genetic disruption of the VHL/HIF system alters skeletal phenotype in all bone cells. PHD and VHL are key regulatory proteins in the HIF pathway whose roles in determining bone mass phenotypes have been well characterized. Combinatorial deletion of PHD isoforms prevent HIF‐α hydroxylation thus allowing HIF‐α isoforms to evade detection and degradation by VHL proteins, resulting in HBM phenotypes.^(^ ^31^ ^)^ Similarly, V*hl* deletion in osteoprogenitors^(^ ^33^ ^)^ and osteoblasts^(^ ^14^, ^34^ ^)^ generated a HBM phenotype due to increased levels of stabilized HIF‐α isoforms that induce osteoanabolic gene transcriptional changes. Thus, stabilized HIF‐α isoforms show osteoanabolic potential. Our studies showed that osteocytic *Vhl* deletion resulted in a HBM phenotype characterized by increases in trabecular and cortical bone microarchitecture.^(^ ^20^ ^)^

To evaluate the distinct effects of HIF‐α isoform accumulation on skeletal phenotype, we used a cre‐loxP system under the control of a 10‐kb‐*Dmp1* promoter to express a degradation resistant HIF‐1α (*Dmp1‐cre*; HIF‐1α cDR) or HIF‐2α (*Dmp1‐cre*; HIF‐2α cDR) mutant protein. HIF‐α mutant protein has a proline to alanine amino acid substitution at the primary hydroxylation site of HIF‐α; preventing its hydroxylation by PHD proteins, allowing it to escape VHL recognition and subsequent proteasomal degradation.^(^ ^21^ ^)^. The 10‐kb *Dmp1‐cre* model is commonly used for osteocyte‐specific loss‐of‐function and gain‐of‐function mutations of a myriad of important skeletal regulatory molecules like RANKL^(^ ^35^ ^)^ and β‐catenin^(^ ^36^ ^)^; however, off‐target effects have been reported on a small percentage of osteoblasts, skeletal muscle, certain bone marrow cells, and in cells of the brain and kidney.^(^ ^37^ ^)^ Despite these drawbacks, the 10‐kb Dmp1‐Cre is a desirable model because it uniquely targets early and late osteocytes; unlike late/mature osteocyte‐specific *Sost‐cre* models.^(^ ^38^ ^)^ Herein we demonstrate that *Dmp1‐cre*; HIF‐1α cDR generated a skeletal phenotype indistinguishable from *cre*‐negative controls (Figs. 3 and 4). Similarly, osteoprogenitor‐specific HIF‐1α cDR mutant protein (*Osx‐cre*; HIF‐1α cDR) generated angiogenic gene expression (*Vegf*) enhancement, but no change in skeletal phenotype.^(^ ^12^ ^)^ These data suggest that HIF‐1α does not play the predominant role in determining skeletal phenotype in osteoprogenitors, osteoblasts, or osteocytes.

Previous studies show that HIF‐2α cDR expression in mesenchymal osteoprogenitors (*Prx1‐cre*; HIF‐2α cDR^+/f^) induced significant increases in *Vegf* and *Opg* expression; however, *Runx2* and *Col1A1* (known osteogenic cell commitment marker) expression remained unaffected; indicating that bone marrow stromal cell (BMSC)‐specific HIF‐2α cDR does not affect BMSC commitment to osteogenic lineage in vitro.^(^ ^12^ ^)^ In vivo, this model showed significant decreases in key osteoblast differentiation genes (*Sp7*, *Ibsp*, *Alp1*), increased osteoprotective *Opg* expression, and enhanced expression of chondrogenic and anti‐osteogenic *Sox9*,^(^ ^39^ ^)^ suggesting that HIF‐2α impairs differentiation of osteoprogenitors into osteoblasts.^(^ ^12^ ^)^ These mice also showed significant decreases in osteoblast number and activity, generating a severe cortical bone thinning phenotype; however, these mice exhibited significantly increased trabecular bone due to increased *Opg* expression in the trabecular compartment.^(^ ^31^ ^)^ Similarly, osteoprogenitor‐specific HIF‐2α cDR (*Osx‐cre*; HIF‐2α cDR) also showed increases in trabecular bone and diminished trabecular and endosteal osteoclasts via increased *Opg* expression.^(^ ^31^ ^)^ In contrast, *Osx‐cre*; HIF‐2α cDR mice did not show a cortical bone phenotype but did show significantly delayed chondrocyte hypertrophy at the growth plate and significant decreases in body weight and limb length.^(^ ^31^ ^)^ These results suggest that HIF‐2α directly inhibits osteoclastogenesis via decoy OPG expression in osteoprogenitors and osteoblasts.^(^ ^12^, ^31^ ^)^

In contrast, in this study we show that mice expressing HIF‐2α cDR in osteocytes had rampant cortical thickening with increased porosity and increased trabecular bone microarchitecture characterized by increased trabecular bone volume and trabecular number. A similar phenotype has been observed in female mice lacking the SOCS3 cytokine signaling suppressor, exhibiting increased trabecular bone and poor corticalization.^(^ ^40^ ^)^ The changes in cortical and trabecular parameters of the HIF‐2α cDR mouse resemble what we have reported in the *Vhl* cKO mouse; however, it is clear that the HIF‐2α cDR phenotype only partially recapitulates the *Vhl* cKO HBM phenotype. The partial recapitulation of the *Vhl* cKO phenotype presented by HIF‐2α cDR mice suggests potential HIF‐α isoform redundancy and/or compensation.

In striking contrast to the *Vhl* cKO, HIF‐2α cDR mice showed significant expansion of marrow stromal tissue in the distal femoral bone marrow compartment at the expense of hematopoietic tissue (Fig. 5). HIF‐2α cDR mice showed ongoing intramembranous bone formation within the bone marrow cavity with numerous bone spicules developing in the expansive stroma and newly embedded osteocytes. In contrast, and as described,^(^ ^20^ ^)^ the *Vhl* cKO distal femoral metaphysis demonstrated thick and abundant trabecularized bone with numerous vasculature‐filled spaces and few bone marrow hematopoietic cells. No increase in bone marrow stroma was evident in *Vhl* cKO or HIF‐1α cDR femurs suggesting that the bone marrow stromal expansion is unique to mice with osteocytic HIF‐2α accumulation. Furthermore, HIF2cDR mice showed a significant increase in osteoclast activity when compared to *Vhl* cKO and all other genotypes (Fig. 6). This suggests that osteocyte‐specific HIF‐2α significantly increases osteoclastogenesis and bone resorption, which is in stark contrast to what has been previously observed in osteoprogenitors and osteoblasts.^(^ ^12^ ^)^ Taken together, these data suggest that increased levels of osteocytic HIF‐2α upregulate osteoclastogenesis resulting in increased bone turnover, increased osteoblastic differentiation, and trabecular bone formation.

The replacement of hematopoietic bone marrow with fibrous stroma we observed in the distal femoral metaphysis of HIF‐2α cDR mice is similar to that of fibrous dysplasia pathophysiology. Previous studies show that fibrous dysplasia is linked to parathyroid hormone related peptide (PTHrP) expression and secretion.^(^ ^41^, ^42^ ^)^ PTHrP is a paracrine regulator of skeletal phenotype that is secreted by osteoblasts and that binds to parathyroid hormone receptors,^(^ ^43^ ^)^ behaving similarly to parathyroid hormone, an endocrine hormone secreted by the parathyroid gland.^(^ ^44^, ^45^ ^)^ PTHrP enhances osteoclastogenesis^(^ ^46^ ^)^ and stimulates bone formation via enhancing osteoblast differentiation and inhibiting osteoblast apoptosis.^(^ ^47^ ^)^ Abnormally elevated secretion of PTHrP from osteoblasts presents in patients with McCune Albright syndrome (MAS), a disease characterized by polyostotic bone dysplasia.^(^ ^48^ ^)^ Further investigation into potential interactions between HIF and PTHrP pathways may explain the similarity in skeletal phenotype and cellularity that we observe in our HIF‐2α cDR mice compared to bone dysplasia in MAS patients.

In summary, osteocytic HIF‐2α cDR but not HIF‐1α cDR produced a HBM skeletal phenotype that partially recapitulated that of *Vhl* cKO, indicating potential HIF‐1α compensation or involvement of HIF‐independent pathways as observed in other *Vhl*‐driven processes.^(^ ^49^, ^50^ ^)^ Striking histological differences in the distal femoral metaphysis of HIF‐2α cDR mice showed evidence of stromal tissue expansion, increased trabecular bone, and new bone formation associated with increased osteoclast number. These data contrast previous HIF‐2α cDR models in mesenchymal progenitors and osteoprogenitors where osteoclastogenesis was significantly decreased. Further studies are required to understand how HIF‐2α accumulation in osteocytes might result in bone marrow stroma—and possibly mesenchymal progenitor—expansion and how osteocytes stimulate osteoclastogenesis in this model.

## Author Contributions

**Sarah V. Mendoza:** Formal analysis; investigation; visualization; writing – original draft; writing – review and editing. **Deepa K. Murugesh:** Methodology; writing – review and editing. **Blaine A. Christiansen:** Supervision; writing – review and editing. **Zoe O. Genetos:** Investigation; writing – review and editing. **Gabriela G. Loots:** Conceptualization; funding acquisition; methodology; resources; supervision; writing – review and editing. **Damian C. Genetos:** Conceptualization; funding acquisition; supervision; writing – original draft; writing – review and editing. **Clare E. Yellowley:** Conceptualization; funding acquisition; project administration; supervision; writing – original draft; writing – review and editing.

## Conflict Of Interest

All authors declare that they have no relevant or material financial interests that relate to the research described in this paper.

### Peer Review

The peer review history for this article is available at https://publons.com/publon/10.1002/jbm4.10724.

## Supporting information


**Appendix S1.** Supplementary Information
Fig. S1.
Fig. S2.
Fig. S3.
Fig. S4.
Fig. S5.

## Data Availability

Data supporting this study's findings are available from the corresponding author upon reasonable request.
