# Supplementary material for: Degradation‐Resistant Hypoxia Inducible Factor‐2α in Murine Osteocytes Promotes a High Bone Mass Phenotype
Source: JBMR Plus. 2023 Feb 17;7(4):e10724. doi: 10.1002/jbm4.10724 (PMC10097640; doi:10.1002/jbm4.10724)

**SUPPLEMENTAL FIGURE LEGENDS**

**Supplemental 1. Examples of region of interest (ROI) measurements utilized for trabecular microarchitecture quantification**. Representative raw microCT transverse images **(A)** and trabecular compartment bone volume fraction ROI **(B)** of metaphyseal trabecular microarchitecture from *cre*-negative, HIF-1α cDR, HIF-2α cDR, and *Vhl* cKO femora

**Supplemental Figure 2. Examples of region of interest (ROI) measurements utilized for cortical microarchitecture quantification**. Representative raw microCT transverse images **(A)**, cortical thickness ROI **(B)**, and medullary area ROI **(C)** of mid-diaphyseal cortical microarchitecture from *cre*-negative, HIF-1α cDR, HIF-2α cDR, and *Vhl* cKO femora.

**Supplemental Figure 3. HIF-1α protein is dramatically increased in HIF-1α cDR mice.** Representative immunohistochemistry staining for HIF-1α (**A**) and images with no primary antibody as controls (**B**) in the diaphyseal cortex of *cre*-negative, HIF-1α cDR, and *Hif1a* cKO mice. Arrows indicate HIF-1α-positive osteocytes.

**Supplemental Figure 4. HIF-2α protein levels are dramatically increased in HIF-2α cDR mice.** Representative immunohistochemistry staining for HIF-2α (**A**) and images with no primary antibody as controls (**B**) in the diaphyseal cortex of *cre*-negative, HIF-2α cDR, and *Hif2a* cKO mice. Arrows indicate HIF-2α-positive embedded or newly embedded osteocytes.

**Supplemental Figure 5. Single HIF-α isoform accumulation does not significantly alter bone marrow adiposity in mice.** Quantification bone marrow adiposity of the distal femoral bone marrow cavity of *cre*-negative (n = 7), HIF-1α cDR (n = 3), HIF-2α cDR (n = 3), and *Vhl* cKO (n = 3) mice. Bars represent mean ± SD; groups with different letters are statistically different from each other.

Supplemental Figure 1


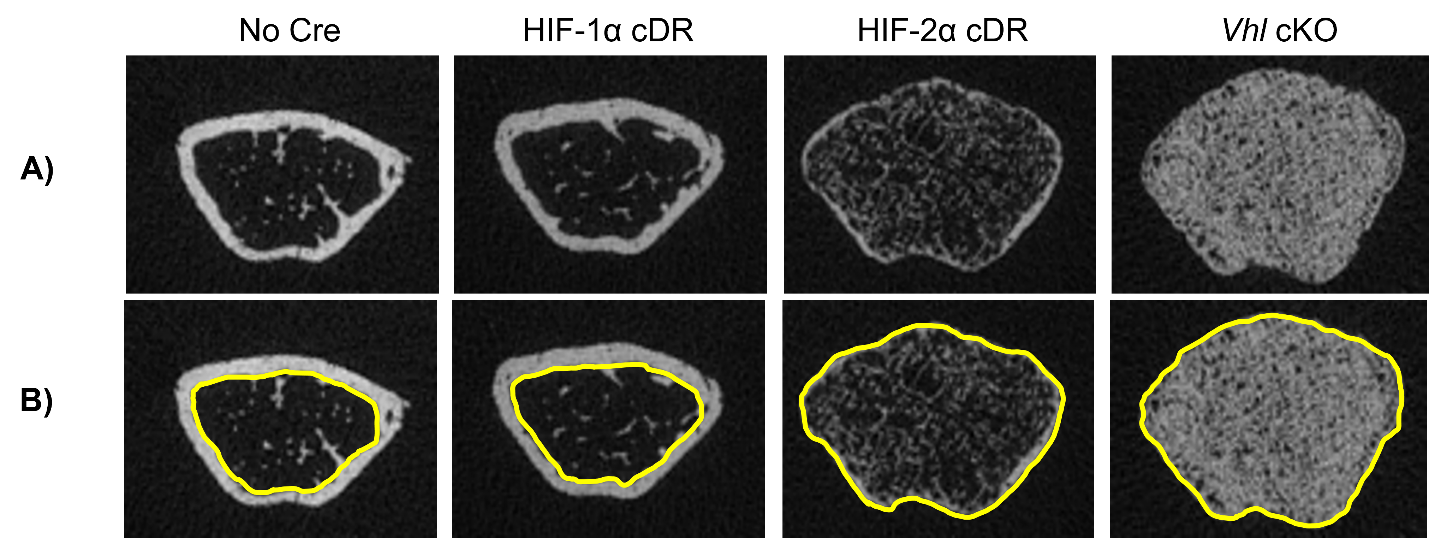


Supplemental Figure 2


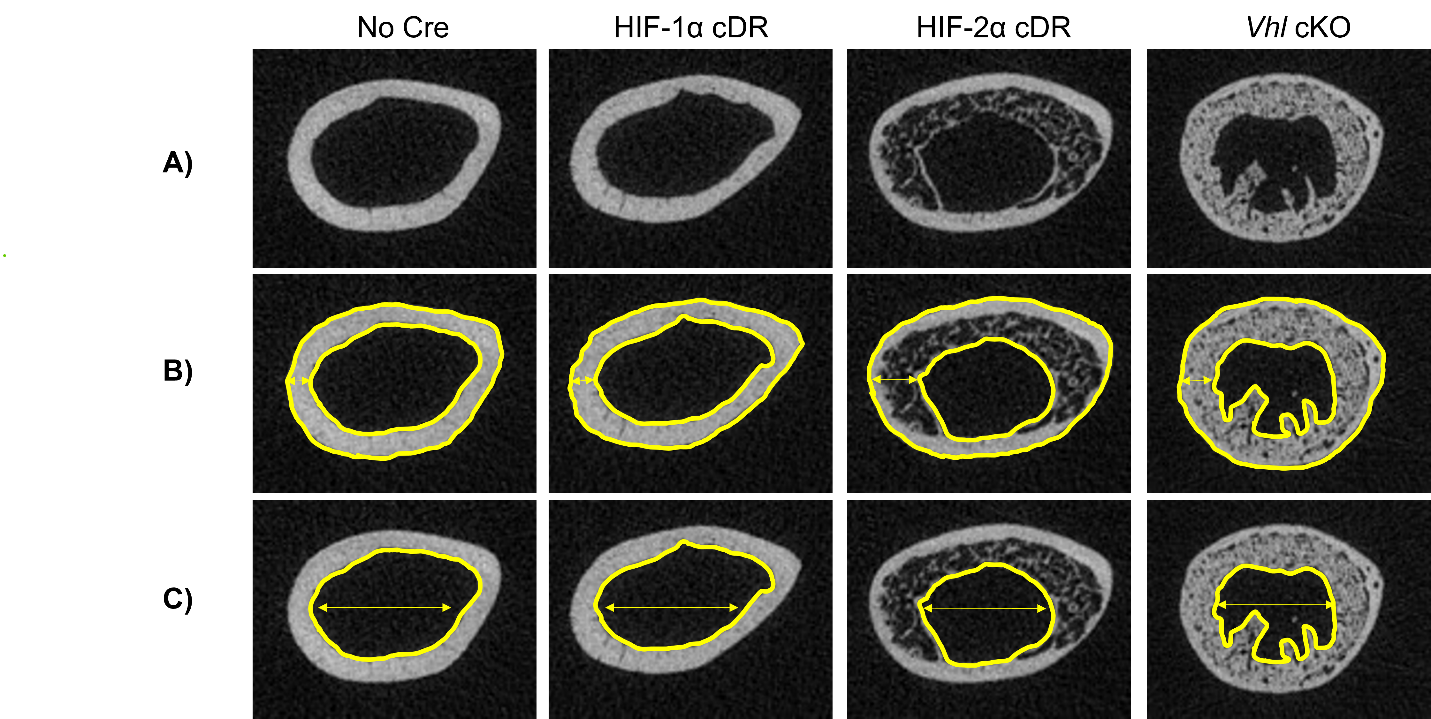


Supplemental Figure 3


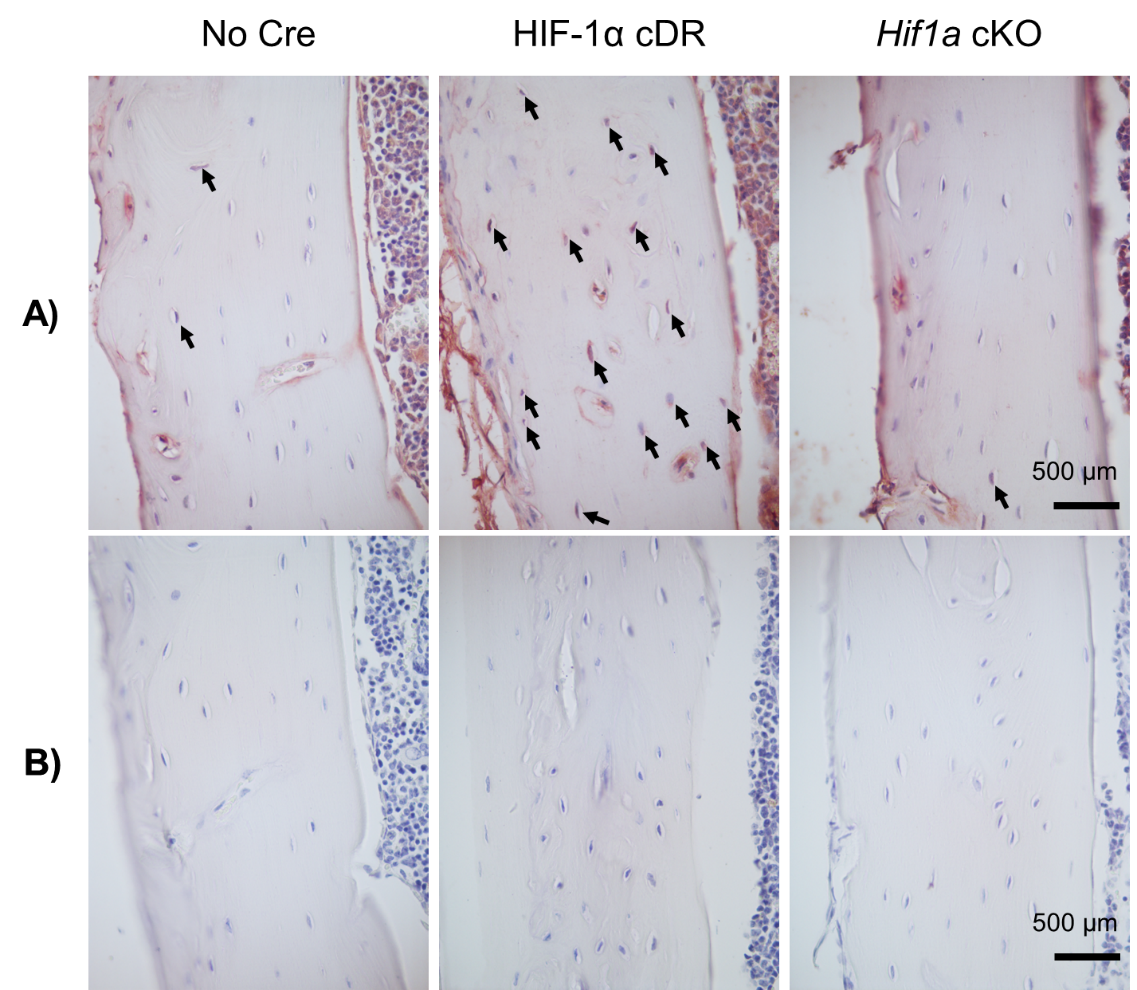


Supplemental Figure 4


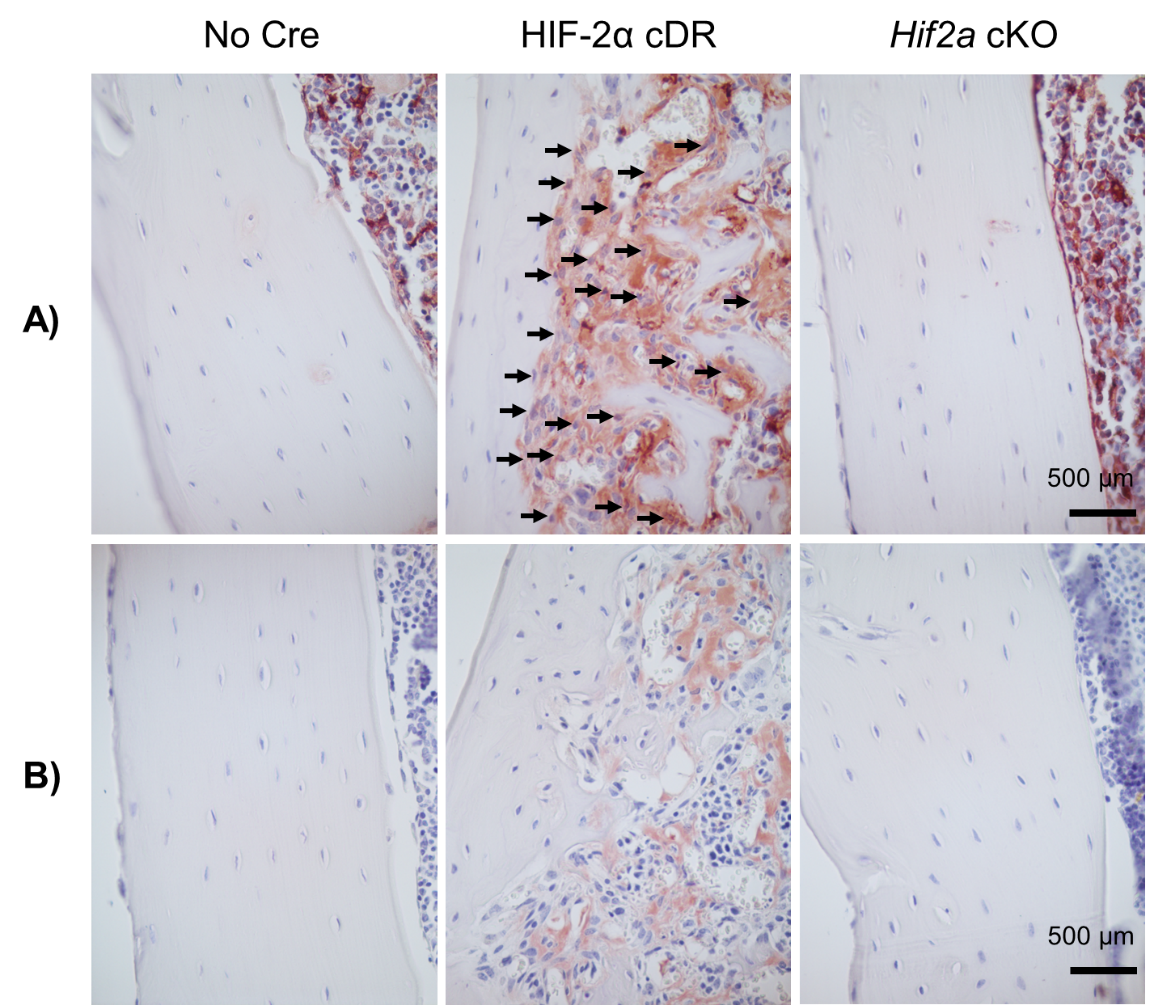


Supplemental Figure 5


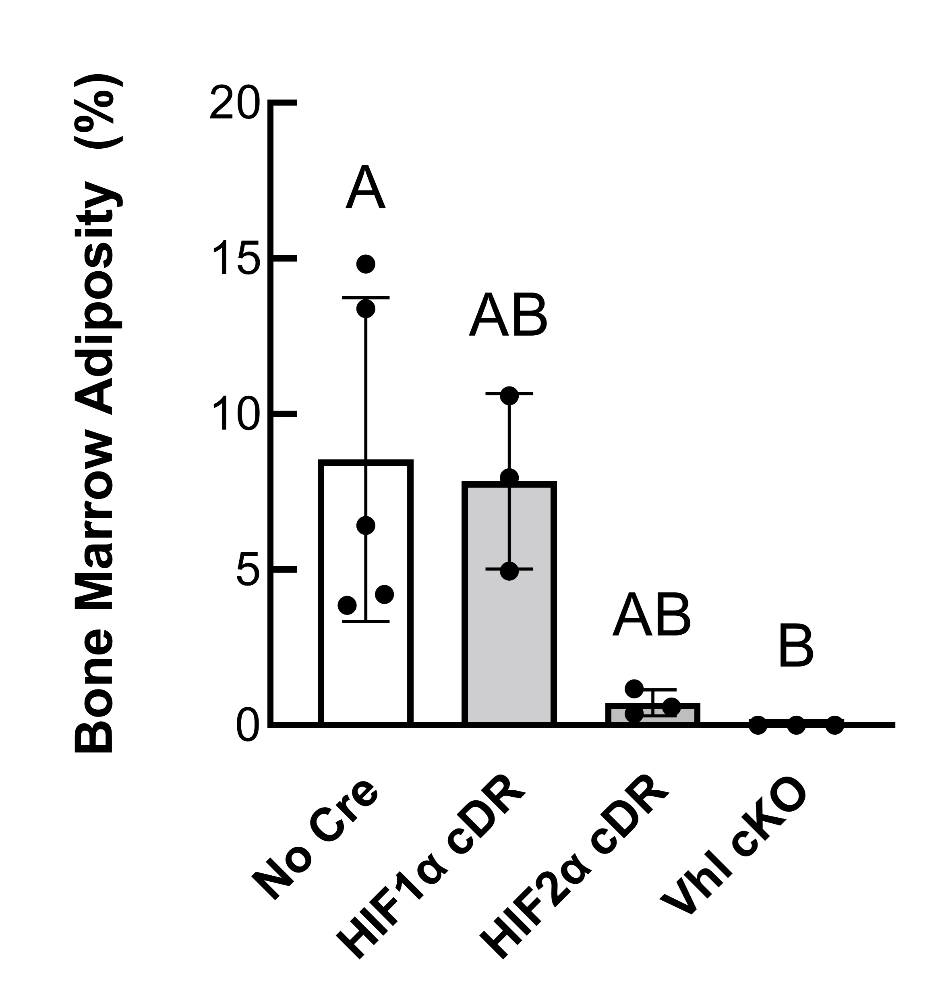

Supplement: Supplementary file 1 — Appendix S1. Supplementary Information Fig. S1. Fig. S2. Fig. S3. Fig. S4. Fig. S5. [file JBM4-7-e10724-s001.docx]
